# Diversity and distribution of the Caddisflies (Insecta: Trichoptera) of Ecuador

**DOI:** 10.7717/peerj.2851

**Published:** 2017-01-12

**Authors:** Blanca Ríos-Touma, Ralph W. Holzenthal, Jolanda Huisman, Robin Thomson, Ernesto Rázuri-Gonzales

**Affiliations:** 1 Facultad de Ingenierías y Ciencias Agropecuarias, Ingeniería Ambiental/Unidad de Biotecnología y Medio Ambiente -BIOMA-, Universidad de las Americas, Campus Queri, Calle José Queri, Quito, Ecuador; 2 Department of Entomology, University of Minnesota—Twin Cities Campus, Saint Paul, MN, United States; 3 Departamento de Entomología, Museo de Historia Natural, Universidad Nacional Mayor de San Marcos, Lima, Peru

**Keywords:** Species richness, Aquatic insects, Neotropics, Caddisflies, Ecuador

## Abstract

**Background:**

Aquatic insects and other freshwater animals are some of the most threatened forms of life on Earth. Caddisflies (Trichoptera) are highly biodiverse in the Neotropics and occupy a wide variety of freshwater habitats. In Andean countries, including Ecuador, knowledge of the aquatic biota is limited, and there is a great need for baseline data on the species found in these countries. Here we present the first list of Trichoptera known from Ecuador, a country that harbors two global biodiversity “hotspots.”

**Methods:**

We conducted a literature review of species previously reported from Ecuador and supplemented these data with material we collected during five recent field inventories from about 40 localities spanning both hotspots. Using species presence data for each Ecuadorian province, we calculated the CHAO 2 species estimator to obtain the minimum species richness for the country.

**Results:**

We recorded 310 species, including 48 new records from our own field inventories for the country. CHAO 2 calculations showed that only 54% of the species have been found. Hydroptilidae and Hydropsychidae were the most species rich families. We report the family Xiphocentronidae for the first time from Ecuador as well as several new records of genera from different families.

**Discussion:**

As in the neighboring Andean countries of Colombia and Peru, it is common to find undescribed species of caddisflies. There are vast areas of Ecuador and the northern Andes that are completely unexplored, and we expect that hundreds of new species are yet to be discovered.

## Introduction

Aquatic ecosystems are among the most threatened on Earth, and the biodiversity they contain, particularly insects, is still largely undiscovered in many parts of the world (Vörösmarty et al., 2010). For example, according to species estimators we only know about 30% of the caddisfly species from the northern Andean region of South America (Venezuela, Colombia, Ecuador, Peru) (RW Holzenthal & B Ríos-Touma, unpublished data). The lack of information on the diversity of species, and their distribution and functional role in aquatic ecosystems, makes predictions of the effects of climate change on these ecosystems and their biota difficult, if not impossible (Holzenthal, Thomson & Ríos-Touma, 2015).

Trichoptera, or caddisflies, are exclusively aquatic in the larval and pupal stages except for a very few terrestrial or semi-terrestrial and brackish-water species and one family whose members are marine (Holzenthal, Thomson & Ríos-Touma, 2015). The members of this order are considered to be biological indicators of good to excellent water quality and are highly sensitive to human disturbance to running waters worldwide (Chang et al., 2014). Currently, there are about 15,000 species described, making Trichoptera the second most diverse monophyletic group of aquatic animals, surpassed only by the clade Diptera: Culicomorpha/Psychodomorpha (Malm, Johanson & Wahlberg, 2013). In Trichoptera, The Neotropical region is the 3rd most species rich in the world with 2100 species recorded as of 2008 after the Oriental and Palearctic regions (De Moor & Ivanov, 2008). In terms of endemism of genera, the Neotropics (115 endemic genera) are second only to the Australasian region (120 endemic genera) (De Moor & Ivanov, 2008)

The Neotropical country of Ecuador hosts an amazing diversity of species, many of them threatened, in two biodiversity “hotspots:” the Tropical Andes and the Tumbes-Choco-Magdalena (Myers et al., 2000). The designation of these hotspots did not include insects or any aquatic biota other than fish. However, the diversity and endemicity of aquatic insects are probably much greater in terms of species numbers than the vertebrate fauna. Considering the importance and sensitivity of aquatic invertebrate biota to changes in habitat, spanning across the watershed, increased knowledge of their taxonomy and biology is urgently needed. Trichoptera are probably one of the best known aquatic groups from the Andes of Ecuador, with some available catalogues of species and their distributions (Flint, Holzenthal & Harris, 1999; Holzenthal & Calor, in press). These baseline data, in addition to the wide range of trophic relationships and microhabitats caddisflies exploit, makes this group ideal for biodiversity and biogeographic studies (Holzenthal, Thomson & Ríos-Touma, 2015). The neighboring countries of Colombia and Peru, have full country or regional Trichoptera checklists (Flint & Reyes, 1991; Flint, 1991; Flint, 1996a; Medellín, Ramírez & Rincón, 2004; Muñoz-Quesada, 2000; Rincón-Hernández, 1999). However, there is no checklist or review of species or their distributions for any aquatic insect order for Ecuador. For these reasons the objectives in this study are to: (1) compile the first list of species of the caddisflies of Ecuador with in-country distribution data from the literature and from our own recent collections; and (2) estimate the total species richness of Ecuadorian caddisflies and define priority areas for future surveys.

## Materials & Methods

To gather species information, we referred to the latest version of the *Catalog of Neotropical Trichoptera* (Holzenthal & Calor, in press). We then searched in the original sources to find more detailed locality information and, especially, the Ecuadorian provinces where the species were recorded. Collections of specimens are recorded in the literature from as early as 1899 (Ulmer, 1905), followed by collections in the early 1900s by Paul Rivet (Navás, 1913) with a large set of collections not appearing until the 1970s by Jeffrey Cohen and Andrea Langley under the Ecuador Peace Corps-Smithsonian Institution Aquatic Insect Survey project which are deposited in the National Museum of Natural History, Smithsonian Institution, Washington, DC, (NMNH). The main taxonomic work on this material was done by O.S. Flint, Jr. (NMNH) during the 1980–90s.

**Figure 1:**
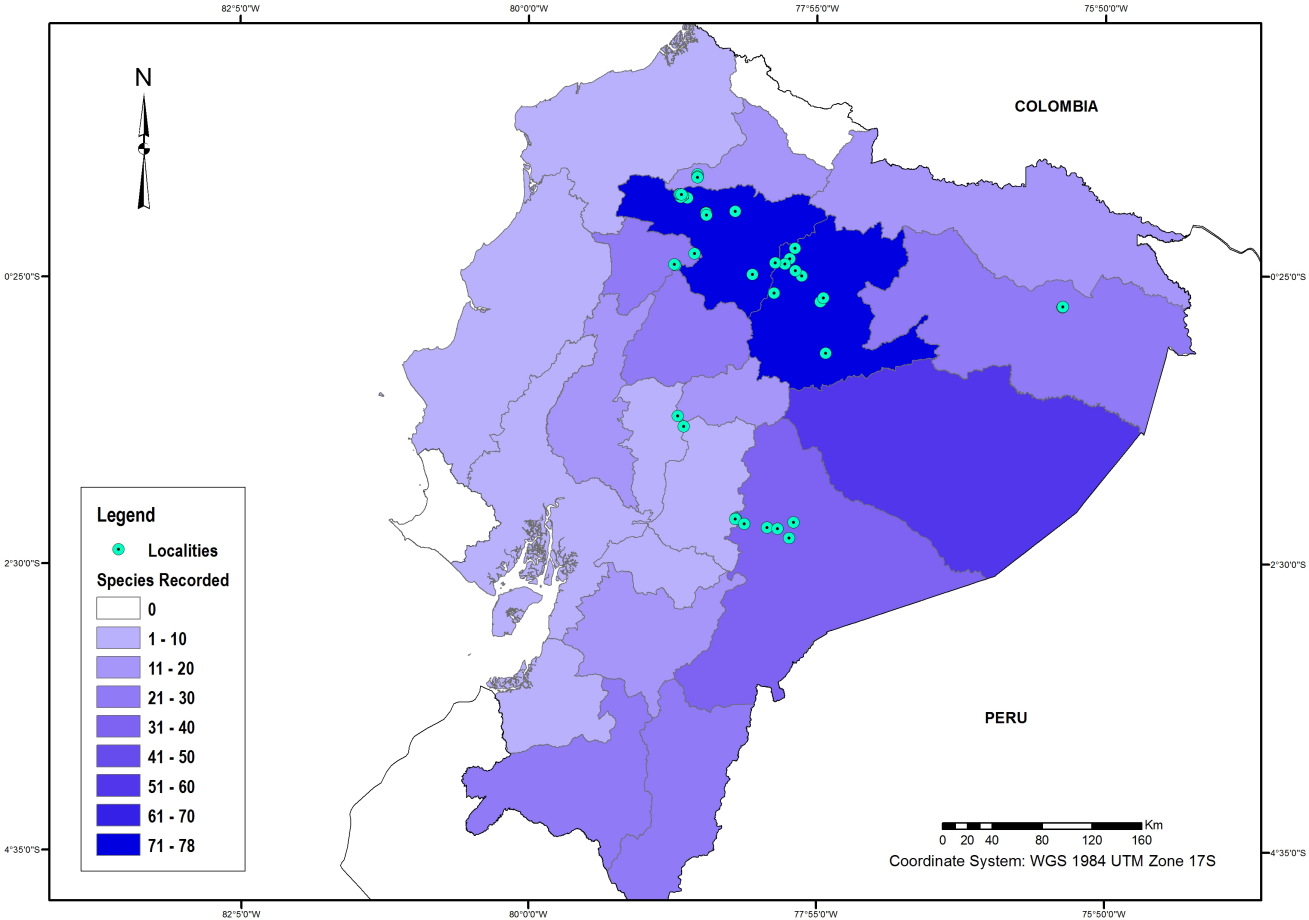
Trichoptera species richness in Ecuador. Caddisfly species per province. Number of species indicated by color intensity. Localities for recent collections indicated by circles.

During the period covered by the literature (1899 until present), several new provinces were created in Ecuador, including Morona Santiago (1952), Napo (1959), Orellana (1998), Santa Elena (2007), Santo Domingo (2007), Sucumbios (1989), and Zamora Chinchipe (1953). Since the historical records do not reflect these new political subdivisions, we tried to relate the locality descriptions to the current province. However, the majority of records did not have exact coordinates and others lacked sufficient locality information to allow us to be certain about the collection site. With the literature information at hand, we made a first list of species (Table 1) that we then compared with our own recent collections from Ecuador from 2010 to 2015 (Table S1). Collection methods used ranged from sweep netting (mainly in páramo locations) to collecting at black lights during the early evening (at all sites). See Blahnik & Holzenthal (2004) and Blahnik, Holzenthal & Prather (2007) for a review of collecting methods, field techniques, and genitalia preparation for adult Trichoptera. We made at least two different collections in each of Chimborazo, Imbabura, Morona Santiago, Napo, Pichincha, and Santo Domingo provinces (for exact locations refer to Table S1 and Fig. 1). Specimens collected in our own research are deposited in the University of Minnesota Insect Collection, St. Paul, Minnesota, USA (UMSP), the Museo Ecuatoriano de Ciencias Naturales, Quito, Ecuador (MECN), the Museo de Ecología Acuática de la Universidad San Francisco de Quito, Ecuador (MUEA-USFQ), and the Museo de Zoología de la Universidad Tecnológica Indoamérica, Quito, Ecuador (MZUTI).

**Table 1:** Trichoptera of Ecuador. Caddisfly species found in Ecuador with their distribution, based on literature records as well as recent collections. Endemic species and new records are indicated.

Species	Province	Endemic	Altitude	Source
**Anomalopsychidae**				
***Contulma***				
*bacula* Holzenthal & Flint, 1995	Napo, Morona Santiago		2,770	Holzenthal & Flint (1995)
*cataracta* Holzenthal & Flint, 1995	Napo, Morona Santiago	E	1,800–3,516	Holzenthal & Flint (1995)
*echinata* Holzenthal & Flint, 1995	Napo		3,690	**NEW RECORD**
*ecuadorensis* Holzenthal & Flint, 1995	Imbabura	E	2,200	Holzenthal & Flint (1995)
*lanceolata* Holzenthal & Flint, 1995	Napo	E	1,260	Holzenthal & Flint (1995)
*paluguillensis* Holzenthal & Ríos-Touma, 2012	Pichincha	E	3,848	Holzenthal & Ríos-Touma (2012)
*papallacta* Holzenthal & Flint, 1995	Napo	E		Holzenthal & Flint (1995)
*penai* Holzenthal & Flint, 1995	Tungurahua, Zamora Chinchipe		1,539–2,000	Holzenthal & Flint (1995)
*spinosa* Holzenthal & Flint, in Flint, 1991	Azuay, Pichincha, Zamora Chinchipe		1,600–3,700	Holzenthal & Flint (1995)
**Atriplectididae**				
***Neoatriplectides***				
sp. (larval record only)	Not known (probable in Ecuador)			Holzenthal (1997)
**Calamoceratidae**				
***Banyallarga***				
*loxana* (Navás), 1934	Azuay, Loja, Zamora Chinchipe		2,000–3,100	Prather (2004)
*penai* Prather, 2004	Loja		2,750	Prather (2004)
*villosa* (Navás), 1934	Loja	E	2,500–2,750	Prather (2004)
***Phylloicus***				
*cressae* Prather, 2003	Napo, Pichincha		950–1,250	Prather (2003)
*elegans* Hogue & Denning, in Denning et al., 1983	Santo Domingo		229	Prather (2003)
*ephippium* Prather, 2003	Tungurahua	E	15,50	Prather (2003)
*fenestratus* Flint, 1974	Napo, Pastaza			Prather (2003)
*lituratus* Banks, 1920	Napo, Pastaza, Santo Domingo		229–1,200	Prather (2003)
*llaviuco* Prather, 2003	Azuay		3,010	Prather (2003)
*paucartambo* Prather, 2003	Napo		1,750	Prather (2003)
*trichothylax* Prather, 2003	Cotopaxi		1,372	Prather (2003)
**Ecnomidae**				
***Austrotinodes***				
*ancylus* Flint & Denning, 1989	Pastaza			Flint & Denning (1989b)
**Glossosomatidae**				
***Mortoniella***				
*angulata* Flint, 1963	Napo, Pichincha	E	3,810	Flint (1963)
*apiculata* Flint, 1963	Napo, Pichincha	E	2,600	Flint (1963)
*aries* (Flint), 1963	Napo, Pastaza, Pichincha		2,440	Flint (1963); this paper
*atenuata* (Flint), 1963	Napo		543	**NEW RECORD**
*bilineata* Ulmer, 1906	Chimborazo, Pichincha, Morona Santiago		550–1,370	Flint (1963)
*chicana* Sykora, 1999	Chimborazo, Pastaza, Napo, Zamora Chinchipe	E	880	Sykora (1999)
*hodgesi* Flint, 1963	Napo, Pichincha	E	4,115	Flint (1963)
*leei* (Flint) 1974	Pichincha		570	**NEW RECORD**
*paralineata* Sykora, 1999	Zamora Chinchipe, Morona Santiago	E	1,340–1,531	Sykora (1999)
*quinuas* Harper & Turcotte, 1985	Azuay	E	3,300	Harper & Turcotte (1985)
*roldani* Flint 1991	Pichincha		570–700	**NEW RECORD**
*santiaga* Sykora, 1999	Morona Santiago	E	2,200	Sykora (1999)
*similis* Sykora, 1999	Santo Domingo	E		Sykora (1999)
*squamata* Sykora, 1999	Napo	E	1,900	Sykora (1999)
*wygodzinskii* (Schmid), 1958	Zamora Chinchipe			Sykora (1999)
***Protoptila***				
*disticha* Flint 1971	Orellana		240–250	**NEW RECORD**
**Helicopsychidae**				
***Helicopsyche***				
*angulata (Feropsyche)* Flint, 1981	Napo			Flint (1981a)
*blahniki (Cochliopsyche)* Johanson, 2003	Pastaza, Napo, Sucumbíos		300	Johanson (2003)
*breviterga (Feropsyche)* Flint 1991	Imbabura, Pichincha		1,312–1,587	**NEW RECORD**
*clara (Cochliopsyche)* (Ulmer), 1905	Pastaza		400	Johanson (2003)
*cochleara (Feropsyche)* Johanson, 1999	Pastaza	E		Johanson (2003)
*cotopaxi (Feropsyche)* Botosaneanu & Flint, 1982	Cotopaxi	E	3,500	Johanson (2002)
*fistulata (Feropsyche)* Flint 1991	Morona Santiago		1,646	**NEW RECORD**
*napoa (Cochliopsyche)* Johanson, 2003	Napo (Sucumbíos), Pastaza	E		Johanson (2003)
*opalescens (Cochliopsyche)* Flint, 1972	Pastaza, Orellana, Sucumbíos			Johanson (2003)
*puyoa (Cochliopsyche)* Johanson, 2003	Pastaza, Orellana, Sucumbíos	E		Johanson (2003)
*vazquezae (Cochliopsyche)* Flint, 1986	Napo, Zamora Chinchipe		950–1,340	Johanson (2003)
*vergelana (Feropsyche)* Ross 1956	Pichincha		570	**NEW RECORD**
*woytkowskii (Feropsyche)* Ross 1956	Morona Santiago. Santo Domingo		1,646	**NEW RECORD**
**Hydrobiosidae**				
***Atopsyche***				
*banksi (Atopsyche)* Ross, 1953	Chimborazo		2,800	Sykora (1991)
*cajas* (unplaced) Harper & Turcotte, 1985	Azuay	E		Harper & Turcotte (1985)
*callosa (Atopsaura)* (Navás), 1924	Azuay, Pichincha, Loja, Santo Domingo, Zamora Chinchipe		550–570, 1,860	Sykora (1991); this paper
*catherinae (Atopsyche)* Harper & Turcotte, 1985	Azuay		3,300	Harper & Turcotte (1985)
*chirihuana (Atopsyche)* Schmid, 1989	Pichincha	E	229	Schmid (1989)
*chirimachaya* (unplaced) Harper & Turcotte, 1985	Azuay		3,300	Harper & Turcotte (1985)
*clarkei (Atopsaura)* Flint, 1963	Morona Santiago		2,200	Sykora (1991)
*copayapu (Atopsyche)* Schmid, 1989	Pichincha, Loja	E	550–1,080	Sykora (1991); this paper
*davidsoni* (unplaced) Sykora, 1991	Bolívar	E	3,420	Sykora (1991)
*flinti* (unplaced) Sykora, 1991	Chimborazo	E	3,500	Sykora (1991)
*incatupac (Atopsyche)* Schmid, 1989	Cotopaxi, El Oro	E	1,780–1,860	Sykora (1991)
*janethae (Atopsyche)* Harper & Turcotte, 1985	Azuay	E	3,300	Harper & Turcotte (1985)
*lobosa (Atopsaura)* Ross & King, 1952	Pichincha		2,807	**NEW RECORD**
*maitacapac (Atopsyche)* Schmid, 1989	Napo	E		Sykora (1991)
*mancocapac (Atopsyche)* Schmid, 1989	Pastaza			Sykora (1991)
*milenae* (unplaced) Sykora, 1991	Bolívar	E		Sykora (1991)
*neolobosa (Atopsaura)* Flint, 1963	Napo, Loja	E	3,200	Flint (1963)
*onorei* (unplaced) Sykora, in Flint et al., 1999	Loja	E		Sykora (1991)
*pachacutec (Atopsyche)* Schmid, 1989	Cotopaxi, El Oro	E		Sykora (1991)
*puharcocha (Atopsaura)* Schmid, 1989	Morona Santiago		2,200	Sykora (1991)
*rawlinsi* (Atopsaura) Sykora, 1991	Loja	E	3,130	Sykora (1991)
*sinchicurac* (Atopsaura) Schmid, 1989	Loja, Zamora Chinchipe	E	1,600–2,500	Schmid (1989)
*tampurima* (Atopsyche) Schmid, 1989	Napo, Zamora Chinchipe		1,420	Schmid (1989) and Sykora (1991)
*tlaloc* (Atopsyche) Schmid, 1989	Azuay	E	2,200–2,400	Schmid (1989) and Sykora (1991)
*vatucra* (Atopsyche) Ross, 1953	Morona Santiago		1,076	**NEW RECORD**
*youngi* (unplaced) Sykora, 1991	Azuay	E	2,600	Sykora (1991)
***Cailloma***				
*lucidula* (Ulmer), 1909	Chimborazo, Pichincha		3,500–3,850	Sykora (1991); this paper.
**Hydropsychidae**				
***Centromacronema***				
*excisum* (Ulmer), 1905	Pichincha		700	Ulmer (1905); this paper
*obscurum* (Ulmer) 1905	Imbabura		1,312	**NEW RECORD**
***Leptonema***				
*album* Mosely, 1933:49	Santo Domingo	E		Oláh & Johanson (2012)
*andrea* Flint, McAlpine & Ross, 1987	Pastaza	E		Flint, McAlpine & Ross (1987)
*cheesmanae* Mosely 1933	Pichincha		570–1,180	**NEW RECORD**
*cinctum* Ulmer, 1905	Bolivar			Ulmer (1905)
*coheni* Flint, McAlpine & Ross, 1987	Cotopaxi	E		Oláh & Johanson (2012)
*divaricatum* Flint, McAlpine & Ross, 1987	Pichincha			Flint, McAlpine & Ross (1987)
*forficulum* Mosely 1933	Pichincha		550–570	**NEW RECORD**
*intermedium* Mosely, 1933	Bolivar, Santo Domingo		600–2,500	Oláh & Johanson (2012)
*janolah* Oláh & Johanson, 2012	Pichincha	E		Oláh & Johanson (2012)
*lojaense* Flint, McAlpine & Ross, 1987	Loja	E		Flint, McAlpine & Ross (1987)
*m&ibulatum* Flint, McAlpine & Ross, 1987	Pastaza, Orellana, Sucumbíos			Flint, McAlpine & Ross (1987)
*mastigion* Flint, McAlpine & Ross, 1987	Los Ríos, Santo Domingo	E	229–600	Flint, McAlpine & Ross (1987); Oláh & Johanson (2012)
*olmos* Olah & Johanson, 2012	Morona Santiago		1,646	**NEW RECORD**
*pseudocinctum* Flint, McAlpine & Ross, 1987	Tungurahua	E	1,280	Flint, McAlpine & Ross (1987)
*rosenbergi* Mosely, 1933	Esmeraldas, Pichincha, Imbabura		600	Flint, McAlpine & Ross (1987); Oláh & Johanson (2012)
*simplex* Mosely, 1933	Loja	E		Flint, McAlpine & Ross (1987)
*sociale* Flint, 2008	Orellana		250	Flint (2008)
*sparsum* (Ulmer), 1905	Pichincha, Napo, Santo Domingo, Orellana, Sucumbíos, Pastaza		400–1,080	Flint, McAlpine & Ross (1987)
*spirillum* Flint, McAlpine & Ross, 1987	Tungurahua, Pastaza, Napo, Imbabura, Morona Santiago		1,300	Flint, McAlpine & Ross (1987)
*stigmosum* Ulmer, 1905	Bolivar			Ulmer (1905)
*trifidum* Flint, McAlpine & Ross, 1987	Napo		510	Flint, McAlpine & Ross (1987)
*viridianum* Navás, 1916	Napo		300–400	Oláh & Johanson (2012)
***Macronema***				
*burmeisteri* Banks, 1924	Sucumbíos		418	Flint (1978)
*fraternum* Banks, 1910	unspecified locality			Flint (1996a)
*hageni* Banks, 1924	Sucumbíos		418	Flint (1978)
*variipenne* Flint & Bueno-Soria, 1979	Cotopaxi, Pichincha		320–550	Flint & Bueno-Soria (1979); this paper
***Macrostemum***				
*ulmeri* (Banks), 1913	unknown			França, Paprocki & Calor (2013)
***Smicridea***				
*acuminata (Rhyacophylax)* Flint, 1974	Morona Santiago, Pichincha, Napo		570–1,076	**NEW RECORD**
*andicola (Rhyacophylax)* Flint, 1991	Pastaza, Tungurahua, Napo			Flint (1991)
*begorba (Rhyacophylax)* Oláh & Johanson, 2012	Napo	E	360	Oláh & Johanson (2012)
*bidactyla (Rhyacophylax)* Flint & Reyes, 1991	El Oro			Flint & Reyes (1991)
*biserrulata (Rhyacophylax)* Flint, 1991	Pichincha, Santo Domingo			Flint (1991); this paper
*bivittata (Smicridea)* (Hagen) 1861	Napo, Santo Domingo, Morona Santiago, Pichincha		600–1,100	Oláh & Johanson (2012); this paper
*curvipenis (Smicridea)* Flint, 1991	Napo		3,600	Flint (1991)
*felsa (Rhyacophylax)* Oláh & Johanson, 2012	Napo	E	400	Oláh & Johanson (2012)
*fogasa (Rhyacophylax)* Oláh & Johanson, 2012	Napo	E	1,660	Oláh & Johanson (2012)
*furesa (Rhyacophylax)* Oláh & Johanson, 2012	Napo		1,100	Oláh & Johanson (2012)
*gemina (Smicridea)* Blahnik, 1995	Cotopaxi, Santo Domingo, Pichincha, Guayas, Esmeraldas, Los Ríos		220–600	Blahnik (1995); this paper
*hajla (Rhyacophylax)* Oláh & Johanson, 2012	Napo	E	400	Oláh & Johanson (2012)
*homora (Rhyacophylax)* Oláh & Johanson, 2012	Napo		400	Oláh & Johanson (2012)
*horga (Smicridea)* Oláh & Johanson, 2012	Pichincha, Santo Domingo	E	550–600	Oláh & Johanson (2012)
*kapara (Rhyacophylax)* Oláh & Johanson, 2012	Morona Santiago		1,076	**NEW RECORD**
*lebena (Rhyacophylax)* Oláh & Johanson, 2012	Napo		1,966	**NEW RECORD**
*medena (Rhyacophylax)* Oláh & Johanson, 2012	Imbabura, Morona Santiago		1,312–1,587	**NEW RECORD**
*murina (Rhyacophylax)* McLachlan, 1871	Pichincha, Napo		570	Flint & Denning (1989a) (unspecified locality); this paper
*nemtompa (Rhyacophylax)* Oláh & Johanson, 2012	Napo		400	Oláh & Johanson (2012)
*nigricans (Smicridea)* Flint, 1991	Tungurahua		1,780	Flint (1991)
*petasata (Rhyacophylax)* Flint, 1981	Pastaza			Flint (1981a)
*polyfasciata (Smicridea)* Martynov, 1912	unspecified locality			Flint (1991)
*probolophora (Rhyacophylax)* Flint, 1991	Morona Santiago		1,531	**NEW RECORD**
*radula (Rhyacophylax)* Flint, 1974	Imbabura		1,312	**NEW RECORD**
*sarkoska (Rhyacophylax)* Oláh & Johanson, 2012	Pichincha, Santo Domingo		1,600	Oláh & Johanson (2012)
*sudara (Rhyacophylax)* Oláh & Johanson, 2012	Pichincha, Santo Domingo		600	Oláh & Johanson (2012)
*tavola (Rhyacophylax)* Oláh & Johanson, 2012	Napo	E	400	Oláh & Johanson (2012)
*tina* Oláh & Johanson, 2012	Santo Domingo		600	Oláh & Johanson (2012)
*truncata (Smicridea)* Flint, 1974	Orellana		240–250	**NEW RECORD**
*varia (Smicridea)* (Banks), 1913	Esmeraldas, Los Ríos, Pichincha, Manabí, Santo Domingo		0–580	Blahnik (1995)
*ventridenticulata (Rhyacophylax)* Flint, 1991	Morona Santiago, Chimborazo, Cotopaxi, Imbabura		800–2,200	Flint (1991)
***Synoestropsis***				Flint (1981a)
*punctipennis* Ulmer, 1905	unspecified locality			
**Hydroptilidae**				
***Acostatrichia***				
*cerna* Oláh & Flint, 2012	Los Ríos	E	250	Oláh & Flint (2012)
*hosulaba* Oláh & Flint, 2012	Pastaza	E		Oláh & Flint (2012)
*kihara* Oláh & Flint, 2012	Napo	E	580	Oláh & Flint (2012)
*pika* Oláh & Flint, 2012	Pichincha, Santo Domingo	E		Oláh & Flint (2012)
*ujasa* Oláh & Flint, 2012	Pastaza	E		Oláh & Flint (2012)
***Anchitrichia***				
*agaboga* Oláh & Flint, 2012	Cotopaxi	E	1,080	Oláh & Flint (2012)
*holzenthali* Oláh & Flint, 2012	Napo	E	950	Oláh & Flint (2012)
*palmatiloba* Flint, 1991	Pichincha, Pastaza, Cotopaxi		330–575	Flint (1991); this paper
***Betrichia***				
*rovatka* Oláh & Johanson, 2011	Pastaza, Napo, Orellana, Sucumbíos			Oláh & Flint (2012)
***Bredinia***				
*dominicensis* Flint, 1968	Esmeraldas, Pichincha			Harris, Holzenthal & Flint (2002)
*espinosa* Harris, Holzenthal & Flint, 2002	Los Ríos, Pichincha, Cotopaxi, Manabí, Guayas			Harris, Holzenthal & Flint (2002)
*manabiensis* Harris, Holzenthal & Flint, 2002	Manabí	E		Harris, Holzenthal & Flint (2002)
*spangleri* Harris, Holzenthal & Flint, 2002	Pastaza, Napo, Cotopaxi	E	350–500	Harris, Holzenthal & Flint (2002)
*venezuelensis* Harris, Holzenthal & Flint, 2002	Pastaza, Napo			Harris, Holzenthal & Flint (2002)
***Byrsopteryx***				
*loja* Harris & Holzenthal, 1994	Zamora Chinchipe	E	2,000	Harris & Holzenthal (1994)
*rayada* Harris & Holzenthal, 1994	Cañar	E	2,910	Harris & Holzenthal (1994)
***Ceratotrichia***				
*felgorba* Oláh & Flint, 2012	Napo	E	580	Oláh & Flint (2012)
*flavicoma* Flint, 1992	Pastaza, Napo, El Oro, Cotopaxi		335	Flint (1992a)
*jobbra* Oláh & Flint, 2012	Manabí, Esmeraldas	E	1,100	Oláh & Flint (2012)
***Costatrichia***				
*noite* Angrisano, 1995	Napo, Sucumbíos			Oláh & Flint (2012)
***Flintiella***				
*astilla* Harris, Flint & Holzenthal, 2002	Napo			Harris, Flint & Holzenthal (2002)
*heredia* Harris, Flint & Holzenthal, 2002	Pastaza			Harris, Flint & Holzenthal (2002)
*pizotensis* Harris, Flint & Holzenthal, 2002	Esmeraldas, Cotopaxi, Napo, Los Rios, Pichincha		340	Harris, Flint & Holzenthal (2002)
*tamaulipasa* Harris, Flint, & Holzenthal 2002	**Orellana**		240	**NEW RECORD**
***Hydroptila***				
*ditalea* Flint, 1968	unspecified locality			Flint & Reyes (1991)
*grenadensis* Flint, 1968	Napo		400	Oláh & Johanson (2011)
*paschia* Mosely, 1937	Pichincha			**NEW RECORD**
*spada* Flint, 1991	Morona Santiago, Pichincha		1,646	**NEW RECORD**
*venezuelensis* Flint, 1981	Napo, Morona Santiago		400	Oláh & Johanson (2011)
***Leucotrichia***				
*fairchildi* Flint, 1970	Los Rios		250	Thomson & Holzenthal (2015)
*forrota* Oláh & Johanson, 2011	Napo, Pastaza			Thomson & Holzenthal (2015)
*fulminea* Thomson & Holzenthal, 2015	Cañar	E	2,910	Thomson & Holzenthal (2015)
*inops* Flint, 1991	Pichincha		1,800	Thomson & Holzenthal (2015)
*pectinata* Thomson & Holzenthal, 2015	Tungurahua	E	1,550	Thomson & Holzenthal (2015)
*riostoumae* Thomson & Holzenthal, 2015	Imbabura	E	1,587	Thomson & Holzenthal (2015)
***Mayatrichia***				
*illobia* Harris & Holzenthal, 1990	Pastaza			Harris & Holzenthal (1990)
***Metrichia***				
*argentinica* (Schmid, 1958)	Pichincha		2,807	**NEW RECORD**
*cuenca* (Harper & Turcotte), 1985	Azuay	E	3,300	Harper & Turcotte (1985)
*patagonica* (Flint), 1983	Pichincha, Morona Santiago		3,848	**NEW RECORD**
*spica* Bueno-Soria & Holzenthal, 2003	Pichincha			**NEW RECORD**
***Neotrichia***				
*biuncifera* Flint, 1974	Morona Santiago		1,076	**NEW RECORD**
*delgadeza* Harris, in Harris & Davenport, 1992	Pastaza	E		Harris & Davenport (1992)
*napoensis* Harris, in Harris & Davenport, 1992	Napo	E		Harris & Davenport (1992)
***Ochrotrichia***				
*ecuatoriana* Bueno-Soria & Santiago-Fragoso, 1992	Pastaza			Bueno-Soria & Santiago-Fragoso (1992)
*puyana* Bueno-Soria & Santiago-Fragoso, 1992	Pastaza	E		Bueno-Soria & Santiago-Fragoso (1992)
*raposa* Bueno-Soria & Santiago-Fragoso, 1992	Esmeraldas, Los Ríos			Bueno-Soria & Santiago-Fragoso (1992)
*yanayacuana* Bueno-Soria & Santiago-Fragoso, 1992	Tungurahua	E	300	Bueno-Soria & Santiago-Fragoso (1992)
***Oxyethira***				
*azteca (Loxotrichia)* (Mosely), 1937	unspecified locality			Flint (1996b)
*apinolada (Oxytrichia)* Holzenthal & Harris, 1992	Pichincha		700	**NEW RECORD**
*circaverna (Dampfitrichia)* Kelley, 1983	Napo			Kelley (1983)
*colombiensis (Tanytrichia)* Kelley, 1983	Los Ríos			Kelley (1983)
*matadero (Dactylotrichia)* Harper & Turcotte, 1985	Azuay	E	3,300	Harper & Turcotte (1985)
*parazteca (Loxotrichia)* Kelley, 1983	Cotopaxi			Kelley (1983)
*parce (Loxotrichia)* (Edwards & Arnold), 1961	Pichincha, Morona Santiago			Flint (1996b); this paper
*quinquaginta (incertae sedis)* Kelley, 1983	Pastaza	E		Kelley (1983)
*scaeodactyla (Dactylotrichia)* Kelley, 1983	Pastaza	E		Kelley (1983)
*simanka* (unplaced) Oláh & Johanson, 2011	Napo	E		Oláh & Johanson (2011)
*tica (Loxotrichia)* Holzenthal & Harris, 1992	unknown			Flint (1996b)
***Rhyacopsyche***				
*benwa* Wasmund & Holzenthal, 2007	Napo		580	Wasmund & Holzenthal (2007)
*bunkotala* Oláh & Johanson, 2011	Napo	E		Oláh & Johanson (2011)
*colubrinosa* Wasmund & Holzenthal, 2007	Cotopaxi, Pastaza, Pichincha, Zamora Chinchipe		330–1,250	Wasmund & Holzenthal (2007)
*hajtoka* Oláh & Johanson, 2011	Pichincha	E		Oláh & Johanson (2011)
*peruviana* Flint, 1975	Pastaza, Zamora Chinchipe			Wasmund & Holzenthal (2007)
*tanylobosa* Wasmund & Holzenthal, 2007	Morona Santiago, Napo, Pastaza, Pichincha, Santo Domingo, Zamora Chinchipe		950–1,531	Wasmund & Holzenthal (2007); this paper
***Zumatrichia***				
*antilliensis* Flint, 1968	Napo		600	Oláh & Flint (2012)
*bevagota* Oláh & Flint, 2012	Cotopaxi	E	1,100	Oláh & Flint (2012)
*corosa* Oláh & Flint, 2012	Cotopaxi	E	1,101	Oláh & Flint (2012)
*fesuka* Oláh & Flint, 2012	Napo	E	580	Oláh & Flint (2012)
*gorba* Oláh & Flint, 2012	Zamora Chinchipe	E	880	Oláh & Flint (2012)
*kerekeda* Oláh & Flint, 2012	Santo Domingo, Pichincha, Cotopaxi, Napo, Manabí, Loja, El Oro, Los Ríos		300–425	Oláh & Flint (2012); this paper
*kisgula* Oláh & Flint, 2012	Napo	E		Oláh & Flint (2012)
*kislaba* Oláh & Flint, 2012	Pastaza	E		Oláh & Flint (2012)
*lapa* Oláh & Flint, 2012	Pastaza	E		Oláh & Flint (2012)
*masa* Oláh & Flint, 2012	Pastaza	E		Oláh & Flint (2012)
*palmara* Flint, 1970	unknown			Flint & Reyes (1991)
*picigula* Oláh & Flint, 2012	Napo, Cotopaxi	E	330–950	Oláh & Flint (2012)
*sima* Oláh & Flint, 2012	Pichincha	E		Oláh & Flint (2012)
**Leptoceridae**				
***Achoropsyche***				
*duodecimpunctata* (Navás), 1916	Orellana		250	This paper, locality previously unspecified
***Amphoropsyche***				
*napo* Holzenthal, 1985	Napo	E		Holzenthal (1985)
*t&ayapa* Holzenthal & Rázuri-Gonzales, 2011	Pichincha	E	1,800	Holzenthal & Rázuri-Gonzales (2011)
***Atanatolica***				
*acuminata* Holzenthal, 1988	Zamora Chinchipe	E		Holzenthal (1988b)
*cotopaxi* Holzenthal, 1988	Cotopaxi, Morona Santiago, Pichincha	E	2,500–3,650	Holzenthal (1988b)
*manabi* Holzenthal, 1988	Manabí, Pichincha	E		Holzenthal (1988b)
***Grumichella***				
*flaveola* (Ulmer), 1911	Loja, Napo, Pastaza, Morona Santiago, Zamora Chinchipe		820–1,800	Holzenthal (1988b)
*trujilloi* Calor & Holzenthal, 2015	Morona Santiago		2,772	**NEW RECORD**
***Nectopsyche***				
*argentata* Flint, 1991	Imbabura, Pichincha		1,180–1,587	Flint (1991)
*gemma* (Müller), 1880	Loja			Navás (1913)
*gemmoides* Flint, 1981	Morona Santiago		1,076	This paper, locality previously unspecified
*maculipennis* Flint 1983	Orellana		240–250	**NEW RECORD**
*muhni* (Navás), 1916	unspecified locality			Flint (1974)
*onyx* Holzenthal 1995	Pichincha		570–700	**NEW RECORD**
*pavida* (Hagen) 1861	Morona Santiago		1,076	**NEW RECORD**
*punctata* (Ulmer), 1905	Pichincha, Napo		570	This paper, locality previously unspecified
*quatuorguttata* (Navas) 1922	Orellana		240–250	**NEW RECORD**
*spiloma* (Ross), 1944	unspecified locality			Flint & Reyes (1991)
*splendida* (Navás), 1917	Orellana		240	Flint (1992b) (unspecified locality); this paper
***Oecetis***				
*acciptrina* Blahnik & Holzenthal, 2014	Los Ríos, Pichincha		550–750	Blahnik & Holzenthal (2014)
*angularis* Blahnik & Holzenthal, 2014	Cotopaxi, Loja, Pichincha, Santo Domingo		300–1,080	Blahnik & Holzenthal (2014)
*campana* Blahnik & Holzenthal, 2014	Zamora Chinchipe, Napo, Pastaza		580–980	Blahnik & Holzenthal (2014)
*constricta* Blahnik & Holzenthal, 2014	Cotopaxi, Guayas, Napo		305–580	Blahnik & Holzenthal (2014)
*excisa* Ulmer, 1907	Orellana, Morona Santiago		240–1,076	**NEW RECORD**
*mexicana* Blahnik & Holzenthal, 2014	Los Ríos		250	Blahnik & Holzenthal (2014)
*protrusa* Blahnik & Holzenthal, 2014	Loja, Pichincha		300–570	Blahnik & Holzenthal (2014); this paper
*pseudoinconspicua* Bueno 1981	Orellana		240–250	**NEW RECORD**
*punctata* (Navás) 1924	Pichincha		570–700	**NEW RECORD**
*punctipennis* (Ulmer), 1905	Orellana		240–250	Quinteiro & Calor (2015) (unspecified locality); this paper
*tumida* Blahnik & Holzenthal 2014	Pichincha		550–575	**NEW RECORD**
***Triaenodes***				
*hodgesi* Holzenthal & Andersen, 2004	Pichincha, Esmeraldas	E	152	Holzenthal & Andersen (2004)
*peruanus* Flint & Reyes, 1991	Santo Domingo		229	Holzenthal & Andersen (2004)
***Triplectides***				
*flintorum* Holzenthal, 1988	Loja		2,000	Holzenthal (1988a)
**Limnephilidae**				
***Anomalocosmoecus***				
*illiesi* (Marlier), 1962	Pichincha		3,848	Flint (1991) (unspecified locality); this paper
**Odontoceridae**				
***Marilia***				
*gigas* Flint, 1991	Pastaza			Flint (1991)
**Philopotamidae**				
***Chimarra***				
*acinaciformis (Curgia)* Flint, 1998	Pastaza	E		Flint (1998)
*centralis (Curgia)* Ross, 1959	Pichincha			Flint (1998)
*coheni (Chimarra)* Blahnik, 1998	Pichincha	E	335	Blahnik (1998)
*creagra (Chimarra)* Flint, 1981	Morona Santiago, Napo		1,076	Flint (1998)
*decimlobata (Chimarra)* Flint, 1991	Imbabura		1,312–1,587	**NEW RECORD**
*didyma (Curgia)* Flint, 1998	Pichincha, Cotopaxi		335–1,100	Flint (1998)
*dolabrifera (Chimarra)* Flint & Reyes, 1991	Pichincha, Cotopaxi, Esmeraldas, Los Ríos		335	Blahnik (1998)
*duckworthi (Chimarra)* Flint, 1967	Pastaza			Blahnik (1998)
*emima (Chimarra)* Ross, 1959	Pichincha, Cotopaxi, Loja, Los Ríos, Santo Domingo		220–550	Blahnik (1998); this paper
*geranoides (Curgia)* Flint, 1998	Pichincha, Pastaza, Tungurahua, Zamora Chinchipe		980–4,200	Flint (1998)
*immaculata (Curgia)* Ulmer, 1911	Napo, Pastaza, Sucumbíos			Flint (1998)
*inflata (Chimarra)* Blahnik, 1998	Napo (Sucumbíos)	E		Blahnik (1998)
*langleyae (Chimarra)* Blahnik, 1998	Napo (Sucumbíos)	E		Blahnik (1998)
*lojaensis (Curgia)* Flint, 1998	Zamora Chinchipe	E	2,000	Blahnik (1998)
*longiterga (Chimarra)* Blahnik & Holzenthal, 1992	Manabí, Pichincha (Santo Domingo)		220	Blahnik (1998)
*macara (Curgia)* Flint, 1998	Loja	E	650	Flint (1998)
*margaritae (Curgia)* Flint, 1991:26	Tungurahua		1,550	Flint (1998)
*munozi (Chimarra)* Blahnik & Holzenthal 1992	Pichincha		570–700	**NEW RECORD**
*onima (Chimarra)* Flint 1991	Pichincha, Santo Domingo		700	**NEW RECORD**
*otuzcoensis (Curgia)* Flint & Reyes, 1991	Pichincha		2,000	Flint (1998)
*pablito (Curgia)* Flint, 1998	Pichincha		570	Flint (1998)
*paracreagra (Chimarra)* Blahnik, 1998	Pastaza, Morona Santiago, Tungurahua		1,280–1,531	Flint (1998)
*peineta (Chimarra)* Blahnik & Holzenthal, 1992	Los Ríos, Santo Domingo, Pichincha		220–550	Blahnik (1998); this paper
*persimilis (Curgia)* Banks, 1920	Los Ríos, Esmeraldas, Pichincha, Loja, Cotopaxi, Manabí		225–600	Flint (1998)
*peruviana (Curgia)* Flint, 1998	Napo, Pastaza		950	Flint (1998)
*prolata (Chimarrita)* Blahnik, 1997	Pastaza	E		Blahnik (1997)
*pumila (Chimarra)* (Banks), 1920	Los Ríos	E		Flint (1998)
*puya (Curgia)* Flint, 1998	Pastaza	E		Flint (1998)
*quadratiterga (Chimarra)* Blahnik, 1998	Zamora Chinchipe	E	980–1,340	Blahnik (1998)
*rafita (Chimarra)* Blahnik, 1998	Pastaza	E	1,000	Blahnik (1998)
*strongyla (Chimarra)* Blahnik, 1998	Pichincha	E	1,100	Blahnik (1998)
*utra (Chimarra)* Blahnik, 1998	Pastaza, Morona Santiago	E	1,076	Blahnik (1998); this paper
*xus (Chimarra)* Blahnik, 1998	Pastaza, Napo			Blahnik (1998)
*zamora (Chimarra)* Blahnik, 1998	Zamora Chinchipe,		980	Blahnik (1998)
***Chimarrhodella***				
*aequatoria* (Navás), 1934	Loja	E		Navás (1934)
*ornata* Blahnik, 2004	Tungurahua	E	1,600	Blahnik (2004)
*ulmeri* (Ross), 1956	Morona Santiago, Pastaza, Tungurahua	E	1,076–1,280	Blahnik (2004)
***Wormaldia***				
*andrea* Muñoz-Quesada & Holzenthal, 2015	Tungurahua	E	1,550	Muñoz-Quesada & Holzenthal (2015)
*araujoi* Muñoz-Quesada & Holzenthal, 2015	Napo	E	640	Muñoz-Quesada & Holzenthal (2015)
*planae* Ross & King, in Ross, 1956	Los Ríos, Pichincha, Santo Domingo		250–1,250	Muñoz-Quesada & Holzenthal (2015); this paper
**Polycentropodidae**				
***Cernotina***				
*cygnea* Flint 1971	Orellana		240–250	**NEW RECORD**
*lobisomem* Santos & Nessimian 2008	Orellana		240–250	**NEW RECORD**
***Cyrnellus***				
*fraternus* (Banks), 1905	unspecified locality			Flint (1982)
*mammillatus* Flint, 1971	Orellana		240–250	Flint (1982) (unspecified locality); this paper
***Polycentropus***				
*altmani* Yamamoto, 1967	unspecified locality			Flint (1981a)
*ceciliae* Flint 1991	Imbabura, Pichincha		1,180–1,587	**NEW RECORD**
*cuspidatus* Flint, 1981	Pastaza			Flint (1981b)
*exsertus* Flint, 1981	Pastaza	E		Flint (1981b)
*joergenseni* Ulmer, 1909b:75	Napo, Morona Santiago		1,646–2,772	Flint & Reyes (1991) (unspecified locality); this paper
*quadricuspidis* Hamilton & Holzenthal, 2005	Zamora Chinchipe	E	2,000	Hamilton & Holzenthal (2005)
*silex* Hamilton & Holzenthal, 2005	Pichincha	E	1,400	Hamilton & Holzenthal (2005)
***Polyplectropus***				
*brborichorum* Chamorro & Holzenthal, 2010	Pastaza	E		Chamorro & Holzenthal (2010)
*buchwaldi* (Ulmer), 1911	unspecified locality	E		Chamorro & Holzenthal (2010)
*ecuadoriensis* Chamorro & Holzenthal, 2010	Sucumbíos, Napo, Pastaza, Cotopaxi			Chamorro & Holzenthal (2010)
*inarmatus* Flint, 1971	Pastaza, Sucumbíos, Orellana		240–1,200	Chamorro & Holzenthal (2010)
*laminatus* (Yamamoto), 1966	El Oro, Pichincha		250–570	Chamorro & Holzenthal (2010)
*puyoensis* Chamorro & Holzenthal, 2010	Pastaza	E		Chamorro & Holzenthal (2010)
*recurvatus* (Yamamoto), 1966	Cotopaxi, Los Ríos		250–330	Chamorro & Holzenthal (2010)
**Xiphocentronidae**				
***Machairocentron***				
*echinatum* (Flint) 1981	Orellana		240–250	**NEW RECORD**
***Xiphocentron***				
sp. (undetermined females only)	Napo		1,966	**NEW RECORD**

From the final list of species (Table 1), we calculated both country richness and province richness. We used the presence of the species per province to calculate an incidence based richness estimator (CHAO 2). This nonparametric species estimator allows for estimation of the potential richness based on the number of observed species, species that are found only in one location, and species that are observed in two locations. Despite its simplicity, a rigorous body of statistical theory demonstrates that CHAO 2 is a robust estimator of minimum richness (Shen, Chao & Lin, 2003) and is more rigorous and performs better in benchmark surveys than extrapolated asymptotic functions or other parametric species richness estimators (Gotelli & Colwell, 2011) with the kind of data used in this study.

## Results

We recorded 310 species of Trichoptera in Ecuador, belonging to 15 families and 52 genera. Literature records contained 264 species for the country (Table 1). Of these, 15 did not have specific locality data, although for nine of them we were able to collect additional specimens representing new locality records. We found 48 species that were not previously reported (Table 1 and Table S1). Pichincha (*n* = 78), Napo (*n* = 75), and Pastaza (*n* = 70) were the provinces with the most species recorded in total. However, since these provinces have been divided into new provinces (see Methods), we could not update all records accurately by province because of incomplete locality descriptions in the historical literature. Accordingly, records for Santo Domingo, Sucumbíos, and Orellana provinces could be diminished. On the other hand, Cañar, Guayas, Bolivar, Chimborazo, El Oro, Manabí, and Esmeraldas provinces have less than 10 species recorded (Fig. 1). Carchi, Santa Elena (which was previously part of Guayas) and Galápagos have no species recorded.

A total of 188 species are only known from one province, and 66 species from three or more provinces. According to the species estimator CHAO 2, the estimated caddisfly richness for the country is 578 species, indicating that only 54% of the Ecuadorian caddisfly fauna is known.

### Family overview

#### Anomalopsychidae

This Neotropical endemic family contains two genera, *Anomalopsyche* and *Contulma*, the latter with 27 species distributed from Costa Rica to Chile and southeastern Brazil. Only *Contulma* is found in Ecuador, where nine species occur (Table 1), of which one is newly recorded from the country.

#### Atriplectididae

This is a very rare family known from only a few widely separated regions (Holzenthal, 1997). The genus is known from Ecuador, but only from one published larval record (Table 1, Holzenthal, 1997) The larvae are unique among all Trichoptera. The head and pronotum are very small and narrow and the anterior portions of the mesothorax are narrow, very elongate, and retractile. Like the adults, they are very rarely collected.

#### Calamoceratidae

This family is cosmopolitan, but most of its 180 species distributed in six genera occur in tropical regions. Two genera, *Banyallarga* and *Phylloicus*, are known for Ecuador with three and eight species respectively (Table 1).

#### Ecnomidae

Only a single genus, *Austrotinodes*, occurs in Ecuador with one recorded species found only in one province (Table 1). In the New World *Austrotinodes* species occur from southern Texas to Chile, with 43 species recorded in the Neotropics (Holzenthal & Calor, in press).

#### Glossosomatidae

The family is cosmopolitan, but only members of the New World, subfamily Protoptilinae occur in the Neotropics. Only the genus *Mortoniella*, with 15 species (Table 1), has been recorded from Ecuador (Holzenthal & Calor, in press). Here we are adding *Protoptila* to the country list with one species (Table 1).

#### Helicopsychidae

All of the species in this cosmopolitan family except one are placed in the genus *Helicopsyche*. Johanson (1998) placed all the Neotropical species in two subgenera, *Feropsyche* and *Cochliopsyche,* both present in Ecuador. Thirteen species of *Helicopsyche* are recorded in Ecuador (with four new records provided in the present study), seven belonging to *Feropsyche* and six to *Cochliopsyche*.

#### Hydrobiosidae

Most of the 52 genera placed in this family occur in the Australian and southern Neotropical regions (Chile and Argentina), a few species are found in the Oriental, Nearctic, and Palearctic regions. Two genera occur in Ecuador (Table 1), *Atopsyche* with 26 species (two new records) and *Cailloma* with 1 species. The genus *Cailloma* occurs only at high altitudes (Sykora, 1991).

#### Hydropsychidae

This is a taxonomically diverse, cosmopolitan family. Five of the six genera known from Ecuador belong to the subfamily Macronematinae (*Centromacronema*, *Leptonema*, *Macronema*, *Macrostemum*, and *Synoestropsis*). *Smicridea* is in the subfamily Smicridiinae. There are 61 species in the family in Ecuador (Table 1) of which we are providing 11 new records. *Smicridea* is by far the most diverse, with 31 species, followed by *Leptonema* with 22. On the other hand, *Macrostemum* and *Synoestropsis* are only known from one species each, both from records with unspecified localities. Adult males of many of the Ecuador species of *Centromacronema*, *Macronema*, and *Macrostemum* swarm during the daytime and do not readily come to lights. After Hydroptilidae, this is the second most species rich family in the country.

#### Hydroptilidae

Microtrichoptera are found around the world and appear to be very diverse in the Neotropics. It is the most diverse family of Trichoptera found in Ecuador, and the most diverse family in the Neotropics. Seven genera and 78 species are recorded for the country, but certainly many more genera and species are yet to be collected. *Zumatrichia* and *Oxyethira* are the most species rich genera in Ecuador. *Betrichia*, *Costatrichia*, and *Mayatrichia* are only known from one species each in the country.

#### Leptoceridae

This is a large, cosmopolitan family of about 50 genera and more than 2,000 species. Eight genera and 33 species are known from Ecuador. The genera present in the country include *Achoropsyche*, *Amphoropsyche, Atanatolica, Grumichella, Nectopsyche, Oecetis, Triaenodes,* and *Triplectides. Nectopsyche* and *Oecetis* are the most species rich genera in the country and *Achoropsyche* and *Triplectides* are each only known from one species.

#### Limnephilidae

This is a large and taxonomically diverse family. Most of its 100 genera and almost 900 species occur in cool lakes and rivers of the northern hemisphere. In the Neotropics they are known only from the higher elevations of Mexico and Central America, the northern and central Andes, and from temperate, southern South America. In Ecuador, the family is known from one species in the genus *Anomalocosmoecus*, from small streams in the high páramo (Table 1).

#### Odontoceridae

About 160 species in 18 genera occur in all faunal regions except the Afrotropical. There are 3 genera in the Neotropics, *Anastomoneura*, *Barypenthus*, and *Marilia*. Only *Marilia* occurs in Ecuador, with one recorded species (Table 1) in the Amazonian region.

#### Philopotamidae

Philopotamids occur in all faunal regions. The Ecuadorian fauna is dominated, both in terms of species diversity and abundance of individuals appearing at lights, by the genus *Chimarra.* Thirty-four *Chimarra* species are known from Ecuador (only 23 have been reported from all of North America). *Chimarrhodella* and *Wormaldia*, with three species each, are also known from Ecuador.

#### Polycentropodidae

Approximately 900 species are known from all faunal regions. Only three genera *Cyrnellus, Polycentropus*, and *Polyplectropus* were previously recorded from Ecuador. We are adding the genus *Cernotina* to the species list with two new records from the Tiputini Biological Research Station. Currently, 18 species in the family are known from Ecuador.

#### Xiphocentronidae

About 170 species are known from the Oriental, Ethiopian, and Neotropical regions (one species extends northward into southern Texas in North America). In the current catalog of Neotropical caddisflies (Holzenthal & Calor, in press) none of the species in this family are recorded from Ecuador. We have collected only three specimens in this family in Ecuador, one of *Machairocentron echinatum* and two unidentifiable female specimens of *Xiphocentron*. This may be because many species are day active and do not come to lights; nevertheless, they are not at all common in the field or in collections.

## Discussion

Hydroptilidae, Hydropsychidae, and Philopotamidae accounted for 58% of the diversity of all Ecuadorian caddisfly species. This pattern is similar to other neotropical countries (Muñoz-Quesada, 2000; Armitage & Cornejo, 2016). Forty-eight new records were added to those that are listed for the country in the current *Catalog of Neotropical Caddisflies* (Holzenthal & Calor, in press), yet the species estimator suggests that 46% of the species present in the country are yet to be discovered. This does not seem far from reality since, for example, Costa Rica has around 460 species recorded (Holzenthal & Calor, in press). Colombia, a country 22 times as large as Costa Rica and four times the size of Ecuador has only 210 species known (Muñoz-Quesada, 2000). Panama, on the other hand has 300 species recorded (Armitage & Cornejo, 2016). The differences between the published records are clearly related to the number of studies and surveys performed in these countries, with Costa Rica and Panama having a long tradition of biodiversity surveys. Considering the diversity of Ecuadorian ecosystems and the fact that some provinces have less than ten records, we are confident that future surveys will find more species. Most of the coastal provinces, including the Ecuadorian Chocó, are understudied and probably harbor species not known from the Amazon or the Andes. These unexplored provinces are priority areas to conduct future collections and surveys. It is important to emphasize that most of the species recorded and added through our own surveys are represented by one or a few individuals.

The land cover loss in the country is high (Sarmiento, 2002; Eva et al., 2004), especially in the Andean and coastal regions. Since several groups of Trichoptera are known to be highly regionally endemic (Previsic et al., 2009), probably some undescribed species are already lost. Also, climate change might play an important role affecting specialist species, such as those found in glacier-fed streams (Jacobsen et al., 2012). Many protected areas in the Andean and coastal regions are located in mountainous areas, protecting only certain species. We have seen that there is an altitudinal segregation for several groups (Helicopsychidae, *Mortoniella*), and altitudinal stratification of caddisfly assemblages has been noted (Rincón-Hernández, 1999; Blinn & Ruiter, 2009). This is also a consideration to take into account for future surveys, and the establishment of protected areas should also address altitudinal zonation.

Many new species of Neotropical caddisflies have been described in recent decades. For example, the number of described species increased from 2,214 in 1999 (Flint et al. 1999) to almost 3,260 species today (Holzenthal & Calor, in press) or an increase of more than 1,000 species in 16 years. Currently, in the material we have collected since 2010 we have tentatively identified around 80 new species and some dozens of species known from unidentifiable females. The description of these new species and the identification of others will increase the number of Ecuadorian species to around 400 with material already in hand.

A list of species is a first step in the chain of knowledge of this diverse and sensitive group of insects. However, other important factors in the protection of species diversity is an understanding of their life history and habitat requirements. Currently, we only have these data for one Ecuadorian caddisfly species, *Contulma paluguillensis* (Holzenthal & Ríos-Touma, 2012). Worldwide, there is a tremendous lack of information on natural history of the world’s biota (Able, 2016). Even in Europe, where almost all the species are described, life-cycle duration, reproduction, and distribution are known for <10% of caddisfly species (Hering et al., 2009). However, this information is crucial to protect and to forecast the effects of climate and land use change on populations and their distributions of this fascinating group of aquatic insects.

##  Supplemental Information

10.7717/peerj.2851/supp-1 Table S1 Caddisfly species by locality from recent collections

## Additional Information and Declarations

### Field Study Permissions

The following information was supplied relating to field study approvals (i.e., approving body and any reference numbers): All the collections were performed under the Ecuadorian research permits: 36-2010-IC-FLO/FAU-DPAP-MA; 0032-FAU-MAE-PNY; 06-2011-ICFAU-DPAI/MA; 005-15-IC-FAU-FLO-DNB/MA issued by the Ministerio del Ambiente de Ecuador.

### Data Availability

The following information was supplied regarding data availability: The raw data has been supplied as a Table S1.
